# 

**Published:** 1888-07

**Authors:** 


					﻿Entered at the Post Office at Austin, Texas, as Second Class Mall Matter.
Vol. IV.
July, 1888.
No. I.
DANIEL'S
MEDICAL
JOURNAL
F. E. DANIEL., M. D., Editor and Publisher, AUSTIN, TEXAS.
ugene Von Boeckmann, Steam Book and Job Printer, Austin, Texas.
				

